# Dichlorido{4-cyclo­hexyl-1-[1-(2-pyridyl-κ*N*)ethyl­idene]thio­semicarbazidato-κ^2^ *N* ^1^,*S*}phenyl­tin(IV)

**DOI:** 10.1107/S1600536810044247

**Published:** 2010-11-06

**Authors:** Md. Abdus Salam, Md. Abu Affan, Fasihuddin B. Ahmad, M. Ibrahim Mohamed Tahir, Edward R. T. Tiekink

**Affiliations:** a Faculty of Resource Science and Technology, Universiti Malaysia Sarawak, 94300 Kota Samarahan, Sarawak, Malaysia; b Faculty of Science, Universiti Putra Malaysia, 43400 UPM Serdang, Selangor, Malaysia; c Department of Chemistry, University of Malaya, 50603 Kuala Lumpur, Malaysia

## Abstract

The Sn^IV^ atom in the title compound, [Sn(C_6_H_5_)(C_14_H_19_N_4_S)Cl_2_], exists within a distorted octa­hedral geometry defined by the *N*,*N*′,*S*-tridentate monodeprotonated Schiff base ligand, two mutually *trans* Cl atoms, and the *ipso*-C atom of the Sn-bound phenyl group; the latter is *trans* to the azo-N atom. The greatest distortion from the ideal geometry is found in the nominally *trans* angle formed by the S and pyridyl-N atoms at Sn [151.03 (4)°]. With the exception of the cyclo­hexyl group (chair form), the Schiff base ligand is almost planar (r.m.s. deviation of non-H and Sn atoms = 0.053 Å). The nearly orthogonal orientation of the Sn-bound phenyl group [N—Sn—C—C torsion angle = 70.8 (5)°] to the planar portion of the Schiff base allows for the formation of significant intra­molecular C—H⋯Cl inter­actions which preclude the Cl atoms from participating in N—H⋯Cl hydrogen bonds. Instead, C—H⋯π contacts, involving methyl­ene H and the Sn-bound phenyl group, lead to the formation of supra­molecular chains that pack in the *bc* plane. Connections between these layers are of the type C—H⋯Cl.

## Related literature

For the structure of the methyl­tin derivative, see: Salam *et al.* (2010 ▶).
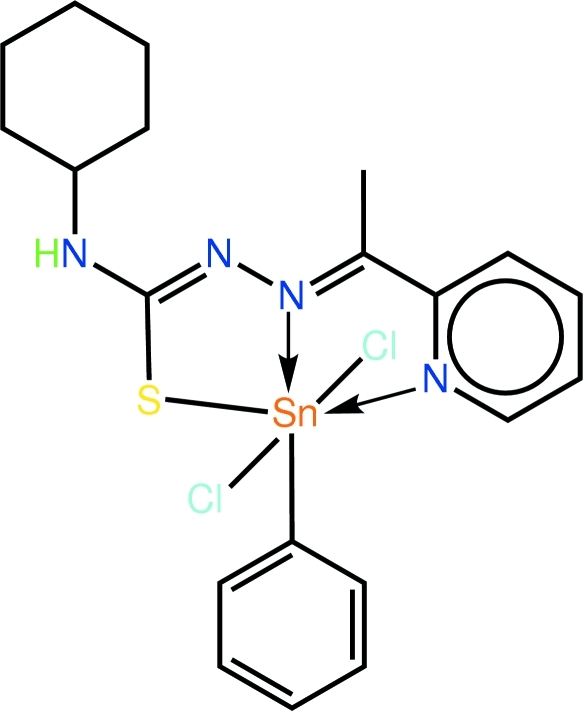


## Experimental

### 

#### Crystal data

[Sn(C_6_H_5_)(C_14_H_19_N_4_S)Cl_2_]
*M* *_r_* = 542.08
Monoclinic, 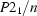

*a* = 11.5213 (2) Å
*b* = 13.3795 (2) Å
*c* = 15.2648 (2) Å
β = 109.630 (2)°
*V* = 2216.30 (6) Å^3^
*Z* = 4
Mo *K*α radiation
μ = 1.50 mm^−1^
*T* = 150 K
0.37 × 0.21 × 0.15 mm

#### Data collection

Area diffractometer
Absorption correction: multi-scan (*CrysAlis RED*; Oxford Diffraction, 2002 ▶) *T*_min_ = 0.730, *T*_max_ = 0.798
23984 measured reflections
3900 independent reflections
3642 reflections with *I* > 2σ(*I*)
*R*_int_ = 0.034

#### Refinement

*R*[*F* ^2^ > 2σ(*F* ^2^)] = 0.018
*wR*(*F* ^2^) = 0.047
*S* = 1.02
3900 reflections
257 parameters
1 restraint
H atoms treated by a mixture of independent and constrained refinement
Δρ_max_ = 0.30 e Å^−3^
Δρ_min_ = −0.45 e Å^−3^

### 

Data collection: *CrysAlis CCD* (Oxford Diffraction, 2002 ▶); cell refinement: *CrysAlis RED* (Oxford Diffraction, 2002 ▶); data reduction: *CrysAlis RED*; program(s) used to solve structure: *SHELXS97* (Sheldrick, 2008 ▶); program(s) used to refine structure: *SHELXL97* (Sheldrick, 2008 ▶); molecular graphics: *ORTEP-3* (Farrugia, 1997 ▶) and *DIAMOND* (Brandenburg, 2006 ▶); software used to prepare material for publication: *publCIF* (Westrip, 2010 ▶).

## Supplementary Material

Crystal structure: contains datablocks global, I. DOI: 10.1107/S1600536810044247/hb5715sup1.cif

Structure factors: contains datablocks I. DOI: 10.1107/S1600536810044247/hb5715Isup2.hkl

Additional supplementary materials:  crystallographic information; 3D view; checkCIF report

## Figures and Tables

**Table 1:** Selected bond lengths (Å)

Sn—Cl1	2.4587 (5)
Sn—Cl2	2.5083 (5)
Sn—S1	2.4768 (5)
Sn—N1	2.2552 (15)
Sn—N2	2.2309 (15)
Sn—C15	2.1552 (17)

**Table 2:** Hydrogen-bond geometry (Å, °) *Cg*1 is the centroid of the C15–C20 benzene ring.

*D*—H⋯*A*	*D*—H	H⋯*A*	*D*⋯*A*	*D*—H⋯*A*
C11—H11*B*⋯*Cg*1^i^	0.99	2.60	3.522 (2)	155
C13—H13*B*⋯Cl1^ii^	0.99	2.80	3.632 (2)	142
C16—H16⋯Cl2	0.95	2.66	3.350 (2)	130
C20—H20⋯Cl1	0.95	2.70	3.363 (2)	127

Symmetry codes: (i) 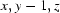; (ii) 
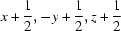
.

**Fig. 1.**
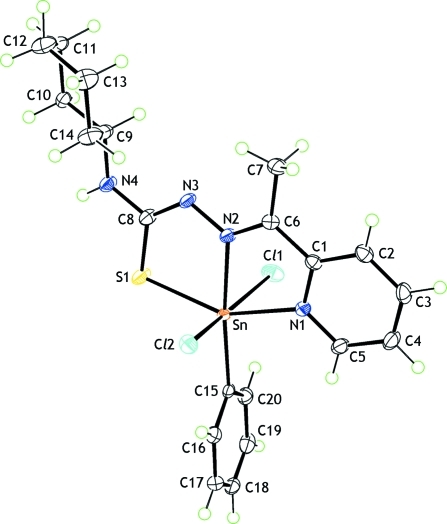
The molecular structure of (I) showing displacement ellipsoids at the 50% probability level.

**Fig. 2.**
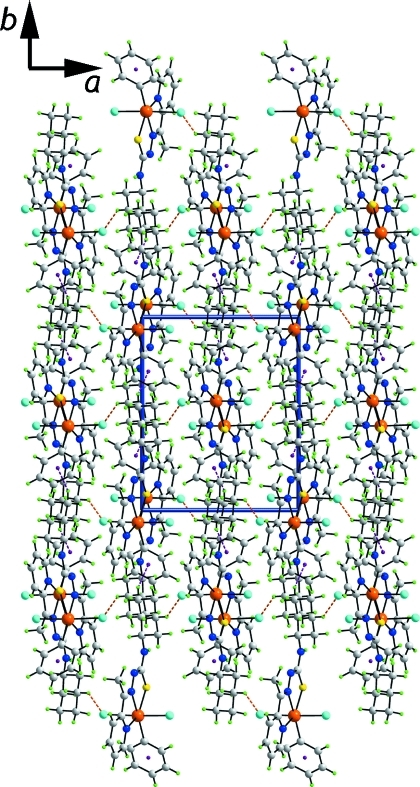
Unit-cell contents shown in projection down the c axis in (I). The C–H···Cl and C–H···π contacts are shown as orange and purple dashed lines, respectively.

## supplementary crystallographic information

### Comment

The title compound, (I), is the phenyltin derivative of the recently described structure of dichlorido{4-cyclohexyl-1-[1-(2-pyridyl-κ*N*)ethylidene]thiosemicarbazidato-κ^2^*N*^1^,*S*}methyltin(IV) (Salam *et al.*, 2010).

The Sn atom in (I), Fig. 1, exists within a six atom CCl_2_N_2_S donor set defined by the tridentate monodeprotonated Schiff base ligand, two mutually *trans* chlorido atoms, and the *ispo*-C atom of the Sn-bound phenyl group which is *trans* to the azo-N atom, Table 1. Distortions from the ideal octahedral geometry are ascribed primarily to the restricted bite distances formed by the Schiff base which results in an angle of 151.03 (4) ° for the nominally *trans* S1—Sn—N1 angle. The disposition of donor atoms resembles that found in the structure of the methyltin derivative (Salam *et al.*, 2010). With the exception of the cyclohexyl group, which adopts a chair conformation, the Schiff base ligand is planar. Thus, the r.m.s. deviation from the least-squares plane through the 15 non-H atoms in the conjugated part of the ligand including the Sn and methine-C15 atoms, *i.e*. Sn,S1,N1–N4,C1–C9, is 0.053 Å. The Sn-bound phenyl group occupies a position almost orthogonal to the chelate rings as seen in the N2—Sn—C15—C16 torsion angle of 70.8 (5) Å.

In the crystal, supramolecular chains along the *b* axis are mediated by C—H···π interactions involving a methylene-H interacting with the Sn-bound phenyl group, Table 1. The chains pack in the *bc* plane and connections between the layers stacked along the *a* axis are of the type C—H···Cl, Fig. 2 and Table 1. The non-participation of the N—H atom in forming a significant intermolecular interaction contrasts the formation of N—H···Cl interactions in the methyltin derivative (Salam *et al.*, 2010). It is noted that the orthogonal orientation of the Sn-bound phenyl group allows for the formation of close intramolecular C—H···Cl contacts, Table 1, which probably deactivate the chlorido atoms from forming significant hydrogen bonds. Further, it is noted that the C—H···π interactions present in (I) involves the Sn-bound phenyl group as as the acceptor, and that these are not possible in the methyltin derivative. Together, these factors explain the absence of significant hydrogen bonding interactions involving the N—H atom.

### Experimental

2-Acetylpyridine-*N*-cyclohexyl thiosemicarbazone (0.28 g, 1.0 mmol) was dissolved in absolute methanol (10 ml) in a Schlenk round bottom flask under a nitrogen atmosphere and stirred for 30 min. Then, a 10 ml me thanolic solution of phenyltin(IV) trichloride (0.302 g, 1.0 mmol) was added drop wise while stirring which resulted in the formation of a yellow solution. The reaction mixture was refluxed for 4 h. and then cooled to room temperature. Yellow micro crystals of (I) were obtained from the slow evaporation of the solution at room temperature. The micro crystals were filtered off, washed with a small amount of cool methanol and dried *in vacuo* over silica gel. Light-yellow crystals were obtained from the slow evaporation of a chloroform/methanol (1/1) solution of (I) held at room temperature. Yield: 0.45 g, 77%: *M*.pt.: 523–525 K. Anal. Calc. for C_20_H_24_Cl_2_N_4_SSn: C, 44.30; H, 4.46; N, 10.33%. Found: C, 44.12; H, 4.27; N, 10.18%.

### Refinement

Carbon-bound H-atoms were placed in calculated positions (C–H = 0.95 to 1.00 Å) and were included in the refinement in the riding model approximation, with *U*_iso_(H) set to 1.2–1.5*U*_equiv_(C). The N-bound H atom was located from a difference map and refined with the distance restraint N–H = 0.88±0.01 Å, and with *U*_iso_(H) = 1.2*U*_eq_(N).

### Crystal data

**Table:**

[Sn(C_6_H_5_)(C_14_H_19_N_4_S)Cl_2_]	*F*(000) = 1088
*M**_r_* = 542.08	*D*_x_ = 1.625 Mg m^−^^3^
Monoclinic, *P*2_1_/*n*	Mo *K*α radiation, λ = 0.71073 Å
Hall symbol: -P 2yn	Cell parameters from 17836 reflections
*a* = 11.5213 (2) Å	θ = 2.4–28.9°
*b* = 13.3795 (2) Å	µ = 1.50 mm^−^^1^
*c* = 15.2648 (2) Å	*T* = 150 K
β = 109.630 (2)°	Prism, orange
*V* = 2216.30 (6) Å^3^	0.37 × 0.21 × 0.15 mm
*Z* = 4	

### Data collection

**Table:**

Area diffractometer	3642 reflections with *I* > 2σ(*I*)
graphite	*R*_int_ = 0.034
ω/2θ scans	θ_max_ = 25.0°, θ_min_ = 2.4°
Absorption correction: multi-scan (*CrysAlis RED*; Oxford Diffraction, 2002)	*h* = −13→13
*T*_min_ = 0.730, *T*_max_ = 0.798	*k* = −15→15
23984 measured reflections	*l* = −18→18
3900 independent reflections	

### Refinement

**Table:**

Refinement on *F*^2^	Primary atom site location: structure-invariant direct methods
Least-squares matrix: full	Secondary atom site location: difference Fourier map
*R*[*F*^2^ > 2σ(*F*^2^)] = 0.018	Hydrogen site location: inferred from neighbouring sites
*wR*(*F*^2^) = 0.047	H atoms treated by a mixture of independent and constrained refinement
*S* = 1.02	*w* = 1/[σ^2^(*F*_o_^2^) + (0.0232*P*)^2^ + 1.3698*P*] where *P* = (*F*_o_^2^ + 2*F*_c_^2^)/3
3900 reflections	(Δ/σ)_max_ = 0.003
257 parameters	Δρ_max_ = 0.30 e Å^−^^3^
1 restraint	Δρ_min_ = −0.45 e Å^−^^3^

### Special details

**Table:**

Geometry. All s.u.'s (except the s.u. in the dihedral angle between two l.s. planes) are estimated using the full covariance matrix. The cell s.u.'s are taken into account individually in the estimation of s.u.'s in distances, angles and torsion angles; correlations between s.u.'s in cell parameters are only used when they are defined by crystal symmetry. An approximate (isotropic) treatment of cell s.u.'s is used for estimating s.u.'s involving l.s. planes.
Refinement. Refinement of *F*^2^ against ALL reflections. The weighted *R*-factor *wR* and goodness of fit *S* are based on *F*^2^, conventional *R*-factors *R* are based on *F*, with *F* set to zero for negative *F*^2^. The threshold expression of *F*^2^ > 2σ(*F*^2^) is used only for calculating *R*-factors(gt) *etc*. and is not relevant to the choice of reflections for refinement. *R*-factors based on *F*^2^ are statistically about twice as large as those based on *F*, and *R*- factors based on ALL data will be even larger.

### Fractional atomic coordinates and isotropic or equivalent isotropic displacement parameters (Å^2^)

**Table:**

	*x*	*y*	*z*	*U*_iso_*/*U*_eq_	
Sn	0.473542 (11)	0.564452 (9)	0.716818 (8)	0.01429 (6)	
Cl1	0.25305 (5)	0.55811 (4)	0.62427 (4)	0.02697 (12)	
Cl2	0.67571 (4)	0.56343 (3)	0.84773 (3)	0.02149 (11)	
S1	0.52690 (6)	0.41751 (4)	0.63924 (4)	0.02665 (13)	
N1	0.39962 (14)	0.63184 (11)	0.82288 (10)	0.0162 (3)	
N2	0.43068 (15)	0.43990 (10)	0.79821 (11)	0.0163 (3)	
N3	0.45002 (15)	0.34299 (11)	0.78079 (11)	0.0186 (3)	
N4	0.51413 (16)	0.23328 (12)	0.69436 (11)	0.0214 (4)	
H4N	0.5435 (19)	0.2243 (16)	0.6499 (11)	0.026*	
C1	0.36701 (17)	0.56662 (13)	0.87825 (13)	0.0160 (4)	
C2	0.31498 (17)	0.60120 (15)	0.94234 (13)	0.0198 (4)	
H2	0.2945	0.5557	0.9827	0.024*	
C3	0.29312 (18)	0.70261 (15)	0.94712 (13)	0.0230 (4)	
H3	0.2559	0.7269	0.9898	0.028*	
C4	0.32570 (19)	0.76806 (15)	0.88952 (14)	0.0242 (4)	
H4	0.3110	0.8377	0.8916	0.029*	
C5	0.38022 (18)	0.72975 (14)	0.82871 (13)	0.0203 (4)	
H5	0.4047	0.7744	0.7898	0.024*	
C6	0.38569 (17)	0.46014 (14)	0.86329 (13)	0.0166 (4)	
C7	0.3507 (2)	0.38016 (14)	0.91856 (14)	0.0240 (4)	
H7A	0.2608	0.3740	0.8978	0.036*	
H7B	0.3817	0.3978	0.9847	0.036*	
H7C	0.3867	0.3164	0.9093	0.036*	
C8	0.49310 (18)	0.32842 (14)	0.71163 (12)	0.0193 (4)	
C9	0.49837 (18)	0.14827 (13)	0.75032 (13)	0.0186 (4)	
H9	0.4180	0.1560	0.7613	0.022*	
C10	0.49480 (18)	0.05184 (13)	0.69666 (14)	0.0179 (4)	
H10A	0.5731	0.0436	0.6841	0.022*	
H10B	0.4268	0.0548	0.6363	0.022*	
C11	0.47560 (19)	−0.03703 (14)	0.75323 (14)	0.0213 (4)	
H11A	0.3950	−0.0304	0.7626	0.026*	
H11B	0.4745	−0.0996	0.7184	0.026*	
C12	0.5780 (2)	−0.04246 (15)	0.84755 (15)	0.0301 (5)	
H12A	0.6577	−0.0555	0.8383	0.036*	
H12B	0.5615	−0.0985	0.8841	0.036*	
C13	0.5861 (2)	0.05525 (15)	0.90122 (15)	0.0305 (5)	
H13A	0.5103	0.0639	0.9173	0.037*	
H13B	0.6571	0.0522	0.9599	0.037*	
C14	0.6013 (2)	0.14412 (15)	0.84392 (14)	0.0272 (5)	
H14A	0.6011	0.2066	0.8786	0.033*	
H14B	0.6816	0.1392	0.8339	0.033*	
C15	0.51354 (17)	0.69707 (13)	0.65203 (12)	0.0151 (4)	
C16	0.62574 (18)	0.74626 (14)	0.68942 (14)	0.0204 (4)	
H16	0.6849	0.7224	0.7453	0.024*	
C17	0.65198 (19)	0.83006 (14)	0.64572 (15)	0.0242 (4)	
H17	0.7292	0.8628	0.6714	0.029*	
C18	0.5663 (2)	0.86581 (14)	0.56524 (14)	0.0245 (5)	
H18	0.5845	0.9231	0.5355	0.029*	
C19	0.45379 (19)	0.81846 (15)	0.52775 (13)	0.0242 (4)	
H19	0.3943	0.8439	0.4728	0.029*	
C20	0.42738 (18)	0.73337 (14)	0.57055 (13)	0.0199 (4)	
H20	0.3506	0.7002	0.5441	0.024*	

### Atomic displacement parameters (Å^2^)

**Table:**

	*U*^11^	*U*^22^	*U*^33^	*U*^12^	*U*^13^	*U*^23^
Sn	0.01933 (9)	0.01020 (8)	0.01541 (8)	−0.00154 (5)	0.00855 (6)	−0.00036 (4)
Cl1	0.0224 (3)	0.0325 (3)	0.0231 (3)	−0.0101 (2)	0.0038 (2)	−0.0018 (2)
Cl2	0.0195 (2)	0.0233 (3)	0.0212 (2)	0.00270 (18)	0.00619 (19)	0.00428 (18)
S1	0.0507 (4)	0.0124 (2)	0.0274 (3)	−0.0002 (2)	0.0270 (3)	−0.0005 (2)
N1	0.0174 (8)	0.0144 (8)	0.0174 (8)	−0.0014 (6)	0.0068 (6)	−0.0016 (6)
N2	0.0219 (9)	0.0122 (8)	0.0161 (8)	−0.0020 (6)	0.0081 (7)	−0.0011 (6)
N3	0.0283 (9)	0.0094 (7)	0.0211 (8)	−0.0005 (7)	0.0122 (7)	−0.0015 (6)
N4	0.0340 (10)	0.0133 (8)	0.0220 (9)	0.0000 (7)	0.0163 (8)	−0.0012 (7)
C1	0.0135 (9)	0.0182 (10)	0.0153 (9)	−0.0033 (7)	0.0037 (7)	−0.0016 (7)
C2	0.0200 (10)	0.0242 (10)	0.0162 (9)	−0.0029 (8)	0.0074 (8)	−0.0020 (8)
C3	0.0213 (10)	0.0274 (11)	0.0226 (10)	−0.0012 (9)	0.0102 (8)	−0.0098 (8)
C4	0.0271 (11)	0.0163 (10)	0.0290 (11)	0.0008 (8)	0.0094 (9)	−0.0064 (8)
C5	0.0240 (10)	0.0144 (9)	0.0228 (10)	−0.0025 (8)	0.0081 (8)	−0.0021 (8)
C6	0.0173 (9)	0.0166 (9)	0.0154 (9)	−0.0020 (8)	0.0048 (8)	0.0004 (7)
C7	0.0318 (11)	0.0207 (10)	0.0248 (10)	−0.0027 (9)	0.0166 (9)	0.0026 (8)
C8	0.0255 (10)	0.0139 (9)	0.0184 (9)	0.0000 (8)	0.0072 (8)	−0.0010 (7)
C9	0.0241 (10)	0.0132 (9)	0.0199 (9)	−0.0015 (8)	0.0094 (8)	0.0003 (8)
C10	0.0208 (10)	0.0145 (9)	0.0198 (10)	0.0009 (8)	0.0085 (8)	−0.0012 (7)
C11	0.0271 (11)	0.0128 (9)	0.0270 (11)	−0.0035 (8)	0.0129 (9)	−0.0024 (8)
C12	0.0369 (13)	0.0184 (10)	0.0322 (12)	−0.0001 (9)	0.0080 (10)	0.0088 (9)
C13	0.0373 (13)	0.0275 (12)	0.0212 (11)	−0.0025 (9)	0.0024 (10)	0.0039 (9)
C14	0.0309 (11)	0.0200 (10)	0.0261 (11)	−0.0066 (9)	0.0035 (9)	−0.0006 (8)
C15	0.0206 (9)	0.0108 (8)	0.0183 (9)	0.0021 (7)	0.0121 (8)	−0.0005 (7)
C16	0.0197 (10)	0.0174 (9)	0.0256 (10)	0.0028 (8)	0.0096 (8)	0.0025 (8)
C17	0.0246 (10)	0.0153 (9)	0.0380 (12)	−0.0016 (8)	0.0173 (9)	0.0002 (9)
C18	0.0379 (12)	0.0142 (9)	0.0322 (11)	0.0037 (9)	0.0261 (10)	0.0057 (8)
C19	0.0347 (12)	0.0226 (10)	0.0197 (10)	0.0100 (9)	0.0150 (9)	0.0064 (8)
C20	0.0227 (10)	0.0194 (10)	0.0196 (9)	0.0017 (8)	0.0096 (8)	−0.0005 (8)

### Geometric parameters (Å, °)

**Table:**

Sn—Cl1	2.4587 (5)	C9—C10	1.521 (2)
Sn—Cl2	2.5083 (5)	C9—C14	1.520 (3)
Sn—S1	2.4768 (5)	C9—H9	1.0000
Sn—N1	2.2552 (15)	C10—C11	1.529 (3)
Sn—N2	2.2309 (15)	C10—H10A	0.9900
Sn—C15	2.1552 (17)	C10—H10B	0.9900
S1—C8	1.7558 (19)	C11—C12	1.526 (3)
N1—C5	1.337 (2)	C11—H11A	0.9900
N1—C1	1.353 (2)	C11—H11B	0.9900
N2—C6	1.294 (2)	C12—C13	1.529 (3)
N2—N3	1.357 (2)	C12—H12A	0.9900
N3—C8	1.323 (2)	C12—H12B	0.9900
N4—C8	1.338 (2)	C13—C14	1.520 (3)
N4—C9	1.470 (2)	C13—H13A	0.9900
N4—H4N	0.863 (19)	C13—H13B	0.9900
C1—C2	1.387 (3)	C14—H14A	0.9900
C1—C6	1.470 (3)	C14—H14B	0.9900
C2—C3	1.386 (3)	C15—C16	1.391 (3)
C2—H2	0.9500	C15—C20	1.392 (3)
C3—C4	1.379 (3)	C16—C17	1.389 (3)
C3—H3	0.9500	C16—H16	0.9500
C4—C5	1.382 (3)	C17—C18	1.378 (3)
C4—H4	0.9500	C17—H17	0.9500
C5—H5	0.9500	C18—C19	1.382 (3)
C6—C7	1.499 (3)	C18—H18	0.9500
C7—H7A	0.9800	C19—C20	1.396 (3)
C7—H7B	0.9800	C19—H19	0.9500
C7—H7C	0.9800	C20—H20	0.9500
			
C15—Sn—N2	172.75 (6)	N4—C9—C14	111.40 (16)
C15—Sn—N1	101.00 (6)	C10—C9—C14	110.28 (16)
N2—Sn—N1	72.01 (5)	N4—C9—H9	108.6
C15—Sn—Cl1	95.95 (5)	C10—C9—H9	108.6
N2—Sn—Cl1	85.12 (4)	C14—C9—H9	108.6
N1—Sn—Cl1	81.94 (4)	C9—C10—C11	109.78 (16)
C15—Sn—S1	107.97 (5)	C9—C10—H10A	109.7
N2—Sn—S1	79.07 (4)	C11—C10—H10A	109.7
N1—Sn—S1	151.03 (4)	C9—C10—H10B	109.7
Cl1—Sn—S1	94.152 (19)	C11—C10—H10B	109.7
C15—Sn—Cl2	94.91 (5)	H10A—C10—H10B	108.2
N2—Sn—Cl2	82.66 (4)	C12—C11—C10	110.94 (16)
N1—Sn—Cl2	84.48 (4)	C12—C11—H11A	109.5
Cl1—Sn—Cl2	164.001 (18)	C10—C11—H11A	109.5
S1—Sn—Cl2	93.632 (18)	C12—C11—H11B	109.5
C8—S1—Sn	95.44 (6)	C10—C11—H11B	109.5
C5—N1—C1	120.18 (16)	H11A—C11—H11B	108.0
C5—N1—Sn	123.46 (13)	C11—C12—C13	110.71 (17)
C1—N1—Sn	116.22 (11)	C11—C12—H12A	109.5
C6—N2—N3	119.00 (15)	C13—C12—H12A	109.5
C6—N2—Sn	119.47 (12)	C11—C12—H12B	109.5
N3—N2—Sn	121.53 (11)	C13—C12—H12B	109.5
C8—N3—N2	115.31 (15)	H12A—C12—H12B	108.1
C8—N4—C9	123.75 (16)	C14—C13—C12	111.01 (18)
C8—N4—H4N	115.6 (15)	C14—C13—H13A	109.4
C9—N4—H4N	120.5 (14)	C12—C13—H13A	109.4
N1—C1—C2	120.13 (16)	C14—C13—H13B	109.4
N1—C1—C6	116.24 (16)	C12—C13—H13B	109.4
C2—C1—C6	123.59 (17)	H13A—C13—H13B	108.0
C1—C2—C3	119.52 (18)	C9—C14—C13	111.42 (16)
C1—C2—H2	120.2	C9—C14—H14A	109.3
C3—C2—H2	120.2	C13—C14—H14A	109.3
C4—C3—C2	119.64 (18)	C9—C14—H14B	109.3
C4—C3—H3	120.2	C13—C14—H14B	109.3
C2—C3—H3	120.2	H14A—C14—H14B	108.0
C3—C4—C5	118.37 (18)	C16—C15—C20	119.14 (17)
C3—C4—H4	120.8	C16—C15—Sn	120.92 (13)
C5—C4—H4	120.8	C20—C15—Sn	119.93 (13)
N1—C5—C4	122.13 (18)	C15—C16—C17	120.47 (18)
N1—C5—H5	118.9	C15—C16—H16	119.8
C4—C5—H5	118.9	C17—C16—H16	119.8
N2—C6—C1	116.06 (16)	C18—C17—C16	120.14 (19)
N2—C6—C7	122.37 (17)	C18—C17—H17	119.9
C1—C6—C7	121.55 (17)	C16—C17—H17	119.9
C6—C7—H7A	109.5	C17—C18—C19	120.10 (18)
C6—C7—H7B	109.5	C17—C18—H18	119.9
H7A—C7—H7B	109.5	C19—C18—H18	119.9
C6—C7—H7C	109.5	C18—C19—C20	120.10 (18)
H7A—C7—H7C	109.5	C18—C19—H19	120.0
H7B—C7—H7C	109.5	C20—C19—H19	120.0
N3—C8—N4	116.01 (17)	C15—C20—C19	120.03 (18)
N3—C8—S1	128.61 (14)	C15—C20—H20	120.0
N4—C8—S1	115.38 (14)	C19—C20—H20	120.0
N4—C9—C10	109.25 (15)		
			
C15—Sn—S1—C8	176.61 (8)	N3—N2—C6—C7	−1.8 (3)
N2—Sn—S1—C8	−1.55 (8)	Sn—N2—C6—C7	177.87 (14)
N1—Sn—S1—C8	−4.91 (11)	N1—C1—C6—N2	−0.4 (3)
Cl1—Sn—S1—C8	−85.76 (7)	C2—C1—C6—N2	177.22 (17)
Cl2—Sn—S1—C8	80.26 (7)	N1—C1—C6—C7	−178.61 (17)
C15—Sn—N1—C5	5.52 (16)	C2—C1—C6—C7	−1.0 (3)
N2—Sn—N1—C5	−176.48 (16)	N2—N3—C8—N4	179.18 (16)
Cl1—Sn—N1—C5	−89.02 (15)	N2—N3—C8—S1	−0.7 (3)
S1—Sn—N1—C5	−173.00 (11)	C9—N4—C8—N3	−3.4 (3)
Cl2—Sn—N1—C5	99.47 (15)	C9—N4—C8—S1	176.47 (14)
C15—Sn—N1—C1	−178.78 (13)	Sn—S1—C8—N3	1.86 (19)
N2—Sn—N1—C1	−0.78 (12)	Sn—S1—C8—N4	−177.98 (14)
Cl1—Sn—N1—C1	86.68 (12)	C8—N4—C9—C10	164.24 (18)
S1—Sn—N1—C1	2.70 (18)	C8—N4—C9—C14	−73.7 (2)
Cl2—Sn—N1—C1	−84.83 (12)	N4—C9—C10—C11	−178.77 (16)
C15—Sn—N2—C6	16.3 (6)	C14—C9—C10—C11	58.5 (2)
N1—Sn—N2—C6	0.57 (14)	C9—C10—C11—C12	−58.4 (2)
Cl1—Sn—N2—C6	−82.53 (14)	C10—C11—C12—C13	56.4 (2)
S1—Sn—N2—C6	−177.72 (15)	C11—C12—C13—C14	−54.7 (3)
Cl2—Sn—N2—C6	87.12 (14)	N4—C9—C14—C13	−179.10 (17)
C15—Sn—N2—N3	−164.1 (4)	C10—C9—C14—C13	−57.6 (2)
N1—Sn—N2—N3	−179.81 (15)	C12—C13—C14—C9	55.6 (3)
Cl1—Sn—N2—N3	97.10 (13)	N2—Sn—C15—C16	70.8 (5)
S1—Sn—N2—N3	1.90 (13)	N1—Sn—C15—C16	86.01 (15)
Cl2—Sn—N2—N3	−93.26 (13)	Cl1—Sn—C15—C16	168.92 (14)
C6—N2—N3—C8	178.28 (17)	S1—Sn—C15—C16	−94.74 (14)
Sn—N2—N3—C8	−1.3 (2)	Cl2—Sn—C15—C16	0.69 (14)
C5—N1—C1—C2	−1.0 (3)	N2—Sn—C15—C20	−110.5 (5)
Sn—N1—C1—C2	−176.82 (14)	N1—Sn—C15—C20	−95.31 (14)
C5—N1—C1—C6	176.77 (17)	Cl1—Sn—C15—C20	−12.40 (14)
Sn—N1—C1—C6	0.9 (2)	S1—Sn—C15—C20	83.93 (14)
N1—C1—C2—C3	2.0 (3)	Cl2—Sn—C15—C20	179.37 (13)
C6—C1—C2—C3	−175.53 (18)	C20—C15—C16—C17	−0.5 (3)
C1—C2—C3—C4	−1.4 (3)	Sn—C15—C16—C17	178.20 (14)
C2—C3—C4—C5	−0.3 (3)	C15—C16—C17—C18	0.6 (3)
C1—N1—C5—C4	−0.8 (3)	C16—C17—C18—C19	0.1 (3)
Sn—N1—C5—C4	174.74 (14)	C17—C18—C19—C20	−1.0 (3)
C3—C4—C5—N1	1.4 (3)	C16—C15—C20—C19	−0.4 (3)
N3—N2—C6—C1	−179.92 (15)	Sn—C15—C20—C19	−179.10 (13)
Sn—N2—C6—C1	−0.3 (2)	C18—C19—C20—C15	1.1 (3)

### Hydrogen-bond geometry (Å, °)

**Table:**

Cg1 is the centroid of benzene ring C15–C20.

**Table:**

*D*—H···*A*	*D*—H	H···*A*	*D*···*A*	*D*—H···*A*
C11—H11B···Cg1^i^	0.99	2.60	3.522 (2)	155
C13—H13B···Cl1^ii^	0.99	2.80	3.632 (2)	142
C16—H16···Cl2	0.95	2.66	3.350 (2)	130
C20—H20···Cl1	0.95	2.70	3.363 (2)	127

Symmetry codes: (i) *x*, *y*−1, *z*; (ii) *x*+1/2, −*y*+1/2, *z*+1/2.
